# Supplementary material for: Training deep quantum neural networks
Source: Nat Commun. 2020 Feb 10;11:808. doi: 10.1038/s41467-020-14454-2 (PMC7010779; doi:10.1038/s41467-020-14454-2)
Supplement: Supplementary file 1 — Supplementary Information [file 41467_2020_14454_MOESM1_ESM.pdf]

Training deep quantum neural networks: Supplementary information

Kerstin Beer,[1,*] Dmytro Bondarenko,[1,†] Terry Farrelly,[1,2,‡] Tobias J. Osborne,[1] Robert Salzmann,[1,3,§] Daniel Scheiermann,[1] and Ramona Wolf[1,¶]

[1] *Institut für Theoretische Physik, Leibniz Universität Hannover, Appelstraße 2, 30167 Hannover, Germany*
[2] *ARC Centre for Engineered Quantum Systems, School of Mathematics and Physics, University of Queensland, Brisbane, QLD 4072, Australia*
[3] *Department of Applied Mathematics and Theoretical Physics, University of Cambridge, Cambridge CB3 0WA, United Kingdom*

[*] kerstin.beer@itp.uni-hannover.de
[†] dimbond@live.com
[‡] farreltc@tcd.ie
[§] rals.salzmann@web.de
[¶] ramona.wolf@itp.uni-hannover.de

SUPPLEMENTARY NOTE 1: UNIVERSALITY AND IMPLEMENTING QUANTUM CHANNELS

It is known (see e.g. [1]) that a neural network composed of classical perceptrons can represent any function. Hence, it is desirable to have the same feature for quantum neural networks.

In order to show universality, we construct a particular network that is capable of universal quantum computation. QNNs turn out to be universal even if each neuron corresponds to just one qubit. However, for more qubits per neuron the construction simplifies and we present separate proofs for single- and dual-rail qubit neurons as well as the most general neurons.

For the case when the perceptron nodes are single qubits, we show that a fully connected network consisting of 4 neurons—two input and two output—can learn any two-qubit unitary V. One possible solution is: the unitary that corresponds to the first output neuron is V on the Hilbert space of input qubits followed by a SWAP on the Hilbert space of the first input and the output qubit, the unitary that corresponds to the second output neuron is a SWAP on the Hilbert space of the second input and the output qubit (see Supplementary Figure 1).

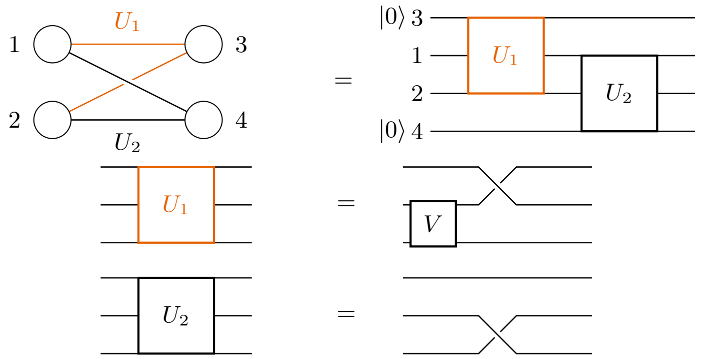

Supplementary Figure 1: Two-qubit unitaries. The fully connected network consisting of two input and two output neurons can learn any two-qubit unitary V.

A QNN built from building blocks of two neurons fully connected to other blocks of two neurons is universal, as two-qubit gates are universal (see e.g. [2]).

For the case when each neuron corresponds to two qubits, we show that each neuron can represent a two-qubit unitary. We number neurons by two indices: neuron (l, j) is the jth neuron in lth layer. Let there be m_l neurons in lth layer. Consider a network where the neuron (l, j) is connected to neurons $(l-1, j)$ and $\left(l+1, j+(-1)^l \mod m_l,\right)$ for all (l, j) and no other connections exist. Suppose that each neuron corresponds qubits labelled by $+$ and $-$, initialised as $|00\rangle$ (as shown in the left picture of Supplementary Figure 2). The action of the neural network on one layer has the form

$$\rho^l = \mathrm{tr}_{l-1}\left(U^l\left(\rho^{l-1}\otimes\left[\bigotimes_{j=1}^{m_l}|00\rangle_{(l,j)}\langle 00|\right]\right)U^{l\dagger}\right), \tag{1}$$

where $U^l = \prod_{j=m_l}^{1} U_j^l$ is a product of each unitary perceptron acting on layers l and $l+1$. For the neuron (l, j), choose

$$U_j^l = V_j^l \ \mathrm{SWAP}\left[(l-1, j, -), \left(l, j-\frac{1+(-1)^l}{2}, +\right)\right] \mathrm{SWAP}\left[(l-1, j, +), \left(l, j+\frac{1-(-1)^l}{2}, -\right)\right],$$

where the SWAP operators act on one qubit in the $l-1$th layer and one qubit that correspond to the neuron (l, j) and V_j^l is a unitary that acts on the qubits of the neuron (l, j). For example, the first swap swaps the $-$ qubit in the neuron (l, j) with the $+$ qubit in neuron $(l, j-(1+(-1)^l)/2)$. Note that for fixed l all swaps commute since they all act on different pairs of qubits. This neural network is equivalent to the quantum circuit of two-qubit gates V_j^l that act on registers number $2j-1$ and $2j$ at the lth time step. This quantum circuit is universal, as two-qubit gates are universal (see e.g. [2]) and SWAP is one of them (see Supplementary Figure 2).

Note that one could easily consider different geometries for the network to allow far away qubits to interact, which may be useful for simulating certain quantum circuits more efficiently.

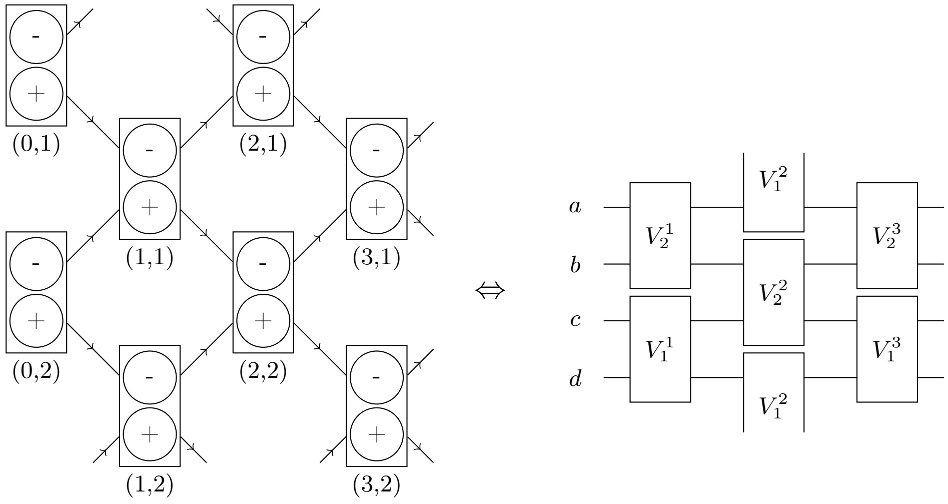

Supplementary Figure 2: Universality of the quantum neural network. The + qubit in neuron $(0,1)$ on the left hand side diagram corresponds to the qubit labelled by a on the right hand side. Similarly, the $(0,2)$ $-$ qubit corresponds to the qubit labelled by b and so on.

It is also straightforward to see that the most general form of a quantum perceptron we allow can implement any quantum channel on the input qubits (or qudits if we are dealing with more general neurons). To see this, look at Equation (1), and suppose that $2m_{l-1} = m_l$. Then it follows from the Stinespring dilation theorem [3] that, because the layer l qubits are in a pure state, we can choose U^l to implement any completely positive map we like on the $l-1$ qubits. Note that the output state lives on the l system as opposed to the $l-1$ systems. This is equivalent to the usual Stinespring protocol by choosing $SU^l = \tilde{U}^l$, where $\tilde{U}^l$ implements the channel we want on the $l-1$ qubits and S swaps these qubits into the first m_{l-1} qubits of the l layer. Of course, this is just a proof of principle. In realistic cases, we would not want to consider generic unitaries U^l that act on $m_{l-1} + m_l$ qubits, but rather we want to choose $U^l = \prod_{j=1}^{m_l} U_j^l$, where each U_j^l acts only on a few qubits. This would be much easier to implement in practice. Then it is an interesting question which channels can be simulated by these more restricted class of perceptrons.

SUPPLEMENTARY NOTE 2: CLASSICAL SIMULATION OF TRAINING THE QNN

In this section, we describe how the simulation of the proposed QNN can be done on a classical computer. The training algorithm is as follows:

I. Initialise:

  I.1 Set $s = 0$.

  I.2 Choose all $U_j^l(0)$ randomly.

II. Feedforward: For each element $\left(|\phi_x^{\text{in}}\rangle, |\phi_x^{\text{out}}\rangle\right)$ in the set of training data, do the following steps: For every layer l, do the following:

  II.1 Tensor the state of the layer to the output state of layer $l-1$, where $\rho_x^{\text{in}} = |\phi_x^{\text{in}}\rangle\langle\phi_x^{\text{in}}|$:

$$\rho_x^{l-1}(s) \otimes |0\ldots0\rangle_l\langle 0\ldots0|$$

  II.2 Apply the unitaries in layer l:

$$U_{m_l}^l(s)U_{m_l-1}^l(s)\ldots U_1^l(s)\left(\rho_x^{l-1}(s) \otimes |0\ldots0\rangle_l\langle 0\ldots0|\right) {U_1^l}^\dagger(s)\ldots {U_{m_l-1}^l}^\dagger(s){U_{m_l}^l}^\dagger(s)$$

  II.3 Trace out layer $l-1$:

$$\rho_x^l(s) = \text{tr}_{l-1}\left(U_{m_l}^l(s)U_{m_l-1}^l(s)\ldots U_1^l(s)\left(\rho_x^{l-1}(s) \otimes |0\ldots0\rangle_l\langle 0\ldots0|\right) {U_1^l}^\dagger(s)\ldots {U_{m_l-1}^l}^\dagger(s){U_{m_l}^l}^\dagger(s)\right).$$

II.4 Store $\rho_x^l(s)$. This step is crucial to efficiently calculate the parameter matrices.

These steps are equivalent to applying the layer-to-layer channels $\mathcal{E}_s^l$ defined in **??** successively to the input state.

III. Update parameters:

III.1 Compute the cost function:

$$C(s) = \frac{1}{N}\sum_{x=1}^{N} \langle \phi_x^{\text{out}} | \rho_x^{\text{out}}(s) | \phi_x^{\text{out}} \rangle$$

III.2 Calculate each parameter matrix $K_j^l(s)$. (How to do this is explained below.)

III.3 Update each perceptron unitary via

$$U_j^l(s+\epsilon) = e^{i\epsilon K_j^l(s)} U_j^l(s).$$

III.4 Update $s = s + \epsilon$.

IV. Repeat steps II. and III. until the cost function has reached its maximum.

To perform the algorithm, we need a formula that allows us to compute the parameter matrices $K_j^l(s)$ to update the perceptron unitaries, which we will derive in the following. For clarity, we omit the superscript that indicates the layer since there is only one layer of unitaries. Furthermore, for the unitaries we omit the dependence on s for reasons of clarity. We derive the formula for the parameter matrices $K_j^l(s)$ as follows: Consider the derivative of the cost function,

$$\frac{dC}{ds} = \lim_{\epsilon \to 0} \frac{C(s+\epsilon) - C(s)}{\epsilon}. \tag{2}$$

The unitaries always act on the current layers, e.g. U_1^2 is actually $U_1^2 \otimes \mathbb{I}^2_{2,3,\ldots m_2}$. Let $\rho_x^{\text{in}} = |\phi_x^{\text{in}}\rangle\langle\phi_x^{\text{in}}|$. The output state at step $s+\epsilon$ is then

$$\begin{aligned}
\rho_x^{\text{out}}(s+\epsilon) = \operatorname{tr}_{\text{in,hidden}} \Big(& e^{i\epsilon K^{\text{out}}_{m_{\text{out}}}(s)} U^{\text{out}}_{m_{\text{out}}}(s)\; e^{i\epsilon K^{\text{out}}_{m_{\text{out}}-1}(s)} U^{\text{out}}_{m_{\text{out}}-1}(s) \ldots e^{i\epsilon K_1^1(s)} U_1^1(s) \left(\rho_x^{\text{in}} \otimes |0\ldots0\rangle_{\text{hidden,out}}\langle 0\ldots 0|\right) \\
& U_1^{1\dagger}(s) e^{-i\epsilon K_1^1(s)} \ldots U^{\text{out}\;\dagger}_{m_{\text{out}}-1}(s) e^{-i\epsilon K^{\text{out}}_{m_{\text{out}}-1}(s)}\; U^{\text{out}\;\dagger}_{m_{\text{out}}}(s) e^{-i\epsilon K^{\text{out}}_{m_{\text{out}}}(s)} \Big) \\
= \rho_x^{\text{out}}(s) + i\epsilon \operatorname{tr}_{\text{in,hidden}} \Big(& K^{\text{out}}_{m_{\text{out}}} U^{\text{out}}_{m_{\text{out}}} \ldots U_1^1(s) \left(\rho_x^{\text{in}} \otimes |0\ldots0\rangle_{\text{hidden,out}}\langle 0\ldots 0|\right) U_1^{1\dagger}(s) \ldots U^{\text{out}\;\dagger}_{m_{\text{out}}}(s) \\
& - U^{\text{out}}_{m_{\text{out}}} \ldots U_1^1(s) \left(\rho_x^{\text{in}} \otimes |0\ldots0\rangle_{\text{hidden,out}}\langle 0\ldots 0|\right) U_1^{1\dagger}(s) \ldots U^{\text{out}\;\dagger}_{m_{\text{out}}}(s) K^{\text{out}}_{m_{\text{out}}}(s) + \ldots \\
& + U^{\text{out}}_{m_{\text{out}}} \ldots K_1^1(s) U_1^1(s) \left(\rho_x^{\text{in}} \otimes |0\ldots0\rangle_{\text{hidden,out}}\langle 0\ldots 0|\right) U_1^{1\dagger}(s) \ldots U^{\text{out}\;\dagger}_{m_{\text{out}}}(s) \\
& - U^{\text{out}}_{m_{\text{out}}} \ldots U_1^1(s) \left(\rho_x^{\text{in}} \otimes |0\ldots0\rangle_{\text{hidden,out}}\langle 0\ldots 0|\right) U_1^{1\dagger}(s) K_1^1(s) \ldots U^{\text{out}\;\dagger}_{m_{\text{out}}}(s) \Big) + \mathcal{O}\left(\epsilon^2\right) \\
= \rho_x^{\text{out}}(s) + i\epsilon \operatorname{tr}_{\text{in,hidden}} \Big(& \Big[K^{\text{out}}_{m_{\text{out}}}(s), U^{\text{out}}_{m_{\text{out}}}(s) \ldots U_1^1(s) \left(\rho_x^{\text{in}} \otimes |0\ldots0\rangle_{\text{hidden,out}}\langle 0\ldots 0|\right) U_1^{1\dagger}(s) \ldots U^{\text{out}\;\dagger}_{m_{\text{out}}}(s) \Big] + \ldots \\
& + U^{\text{out}}_{m_{\text{out}}}(s) \ldots U_2^1(s) \Big[K_1^1(s), U_1^1(s) \left(\rho_x^{\text{in}} \otimes |0\ldots0\rangle_{\text{hidden,out}}\langle 0\ldots 0|\right) U_1^{1\dagger}(s) \Big] U_2^{1\dagger}(s) \ldots U^{\text{out}\;\dagger}_{m_{\text{out}}}(s) \Big) + \mathcal{O}\left(\epsilon^2\right).
\end{aligned}$$

The derivative of the cost function up to first order in ϵ can then be written as

$$\begin{aligned}
\frac{dC(s)}{ds} &= \lim_{\epsilon\to 0}\frac{C(s+\epsilon)-C(s)}{\epsilon}\\
&= \lim_{\epsilon\to 0}\frac{C(s)+\frac{i\epsilon}{N}\sum_x \langle\phi_x^{\text{out}}|\left(\rho_x^{\text{out}}(s+\epsilon)\right)|\phi_x^{\text{out}}\rangle - C(s)}{\epsilon}\\
&= \frac{1}{N}\sum_x \operatorname{tr}\Big(\mathbb{I}_{\text{in,hidden}}\otimes|\phi_x^{\text{out}}\rangle\langle\phi_x^{\text{out}}|\,\Big(\Big[iK^{\text{out}}_{m_{\text{out}}}(s),U^{\text{out}}_{m_{\text{out}}}(s)\dots U_1^1(s)\left(\rho_x^{\text{in}}\otimes|0\dots0\rangle_{\text{hidden,out}}\langle 0\dots 0|\right)U_1^{1\dagger}(s)\\
&\quad \dots U^{\text{out}\dagger}_{m_{\text{out}}}(s)\Big]+\dots+U^{\text{out}}_{m_{\text{out}}}(s)\dots U_2^1(s)\left[iK_1^1(s),U_1^1(s)\left(\rho_x^{\text{in}}\otimes|0\dots0\rangle_{\text{hidden,out}}\langle 0\dots0|\right)U_1^{1\dagger}(s)\right]\\
&\quad U_2^{1\dagger}(s)\dots U^{\text{out}\dagger}_{m_{\text{out}}}(s)\Big)\Big)\\
&= \frac{1}{N}\sum_x \operatorname{tr}\Big(\underbrace{\left[U^{\text{out}}_{m_{\text{out}}}(s)\dots\left(\rho_x^{\text{in}}\otimes|0\dots0\rangle_{\text{hidden,out}}\langle0\dots0|\right)\dots U^{\text{out}\dagger}_{m_{\text{out}}}(s),\mathbb{I}_{\text{in,hidden}}\otimes|\phi_x^{\text{out}}\rangle\langle\phi_x^{\text{out}}|\right]}_{\equiv M^{\text{out}}_{m_{\text{out}}}(s)} iK^{\text{out}}_{m_{\text{out}}}(s)+\dots\\
&\quad +\underbrace{\left[U_1^1(s)\left(\rho_x^{\text{in}}\otimes|0\dots0\rangle_{\text{hidden,out}}\langle0\dots0|\right)U_1^{1\dagger}(s),U_2^{1\dagger}(s)\dots U^{\text{out}\dagger}_{m_{\text{out}}}(s)\left(\mathbb{I}_{\text{in+hidden}}\otimes|\psi_x\rangle\langle\psi_x|\right)U^{\text{out}}_{m_{\text{out}}}(s)\dots U_2^1(s)\right]}_{\equiv M_1^1(s)}\\
&\quad iK_1^1(s)\Big)\\
&= \frac{i}{N}\sum_x \operatorname{tr}\left(M^{\text{out}}_{m_{\text{out}}}(s)K^{\text{out}}_{m_{\text{out}}}(s)+\ \dots\ +M_1^1(s)K_1^1(s)\right).
\end{aligned}\tag{3}$$

We will parametrise the parameter matrices as

$$K_j^l(s)=\sum_{\alpha_1,\alpha_2,\dots,\alpha_{m_{l-1}},\beta}K^l_{j,\alpha_1,\dots,\alpha_{m_{l-1}},\beta}(s)\left(\sigma^{\alpha_1}\otimes\ \dots\ \otimes\sigma^{\alpha_{m_{l-1}}}\otimes\sigma^{\beta}\right),$$

where the α_i denote the qubits in the previous layer and β denotes the current qubit in layer l. As described in the example, to reach the maximum of the cost function as a function of the parameters *fastest*, we maximize $\frac{dC}{ds}$. Since this is a linear function, the extrema are at $\pm\infty$. To ensure that we get a finite solution, we introduce a Lagrange multiplier $\lambda\in\mathbb{R}$. Hence, to find K_j^l we have to solve the following maximization problem:

$$\begin{aligned}
&\max_{K^l_{j,\alpha_1,\dots,\beta}}\left(\frac{dC(s)}{ds}-\lambda\sum_{\alpha_i,\beta}K^l_{j,\alpha_1,\dots,\beta}(s)^2\right)\\
&\quad=\max_{K^l_{j,\alpha_1,\dots,\beta}}\left(\frac{i}{N}\sum_x\operatorname{tr}\left(M^{\text{out}}_{m_{\text{out}}}(s)K^{\text{out}}_{m_{\text{out}}}(s)+\ \dots\ +M_1^1(s)K_1^1(s)\right)-\lambda\sum_{\alpha_1,\dots,\beta}K^l_{j,\alpha_1,\dots,\beta}(s)^2\right)\\
&\quad=\max_{K^l_{j,\alpha_1,\dots,\beta}}\left(\frac{i}{N}\sum_x\operatorname{tr}_{\alpha_1,\dots,\beta}\left(\operatorname{tr}_{\text{rest}}\left(M^{\text{out}}_{m_{\text{out}}}(s)K^{\text{out}}_{m_{\text{out}}}(s)+\ \dots\ +M_1^1(s)K_1^1(s)\right)\right)-\lambda\sum_{\alpha_1,\dots,\beta}K^l_{j,\alpha_1,\dots,\beta}(s)^2\right).
\end{aligned}$$

Note that rest in tr_{rest} refers to the complement of $\{\alpha_1,\dots,\beta\}$. Taking the derivative with respect to $K^l_{j,\alpha_1,\dots,\beta}$ yields

$$\frac{i}{N}\sum_x\operatorname{tr}_{\alpha_1,\dots,\beta}\left(\operatorname{tr}_{\text{rest}}\left(M_j^l(s)\right)\left(\sigma^{\alpha_1}\otimes\ \dots\ \otimes\sigma^{\beta}\right)\right)-2\lambda K^l_{j,\alpha_1,\dots,\beta}(s)=0,$$

hence,

$$K^l_{j,\alpha_1,\dots,\beta}(s)=\frac{i}{2N\lambda}\sum_x\operatorname{tr}_{\alpha_1,\dots,\beta}\left(\operatorname{tr}_{\text{rest}}\left(M_j^l(s)\right)\left(\sigma^{\alpha_1}\otimes\ \dots\ \otimes\sigma^{\beta}\right)\right)$$

This yields the matrix

$$\begin{aligned}
K_j^l(s) &= \sum_{\alpha_1,\ldots,\beta} K^l_{j,\alpha_1,\ldots,\beta}(s)\left(\sigma^{\alpha_1}\otimes\ \ldots\ \otimes\sigma^{\beta}\right)\\
&= \frac{i}{2N\lambda}\sum_{\alpha_1,\ldots,\beta}\sum_x \mathrm{tr}_{\alpha_1,\ldots,\beta}\left(\mathrm{tr}_{\text{rest}}\left(M_j^l(s)\right)\left(\sigma^{\alpha_1}\otimes\ \ldots\ \otimes\sigma^{\beta}\right)\right)\left(\sigma^{\alpha_1}\otimes\ \ldots\ \otimes\sigma^{\beta}\right)\\
&= \frac{2^{n_{\alpha_1,\ldots,\beta}}i}{2N\lambda}\sum_x \mathrm{tr}_{\text{rest}}\left(M_j^l(s)\right),
\end{aligned}$$

with

$$\begin{aligned}
M_j^l(s) = \Big[&U_j^l(s)U_{j-1}^l(s)\ldots U_1^1(s)\ \left(\rho_x^{\text{in}}\otimes|0\ldots0\rangle_{\text{hidden,out}}\langle 0\ldots0|\right)U_1^{1\dagger}(s)\ldots U_{j-1}^{l\ \dagger}(s)U_j^{l\dagger}(s),\\
&U_{j+1}^{l\ \dagger}(s)\ldots U_{m_{\text{out}}}^{\text{out}\ \dagger}(s)\left(\mathbb{I}_{\text{in,hidden}}\otimes|\phi_x^{\text{out}}\rangle\langle\phi_x^{\text{out}}|\right)U_{m_{\text{out}}}^{\text{out}}(s)\ldots U_{j+1}^l(s)\Big].
\end{aligned}$$

Note that in the paper, we have introduced the learning rate η, which is related to lambda by $\eta = 1/\lambda$ and referred to it as the *learning rate*.

Now we describe how the channel structure of the feedforward process can be exploited to efficiently train the QNN. Consider a network with L hidden layers and a set of N pairs of training data $\left(|\phi_x^{\text{in}}\rangle, |\phi_x^{\text{out}}\rangle\right)$. As described in the previous sections, the general output state of the network at step s is

$$\rho_x^{\text{out}}(s) = \mathcal{E}_s^{\text{out}}\left(\mathcal{E}_s^L\left(\ldots\mathcal{E}_s^2\left(\mathcal{E}_s^1\left(\rho_x^{\text{in}}\right)\right)\ldots\right)\right)$$

with the channel acting on layer $l-1$ and l being

$$\mathcal{E}_s^l\left(X^{l-1}\right) = \mathrm{tr}_{l-1}\left(U_{m_l}^l(s)\ldots U_1^l(s)\left(X^{l-1}\otimes|0\ldots0\rangle_l\langle0\ldots0|\right)U_1^{l\dagger}(s)\ldots U_{m_l}^{l\ \dagger}(s)\right), \tag{4}$$

where m_l is the number of perceptrons in layer l.

This network structure provides a way to compute the derivative of the cost function that is similar to the back-propagation algorithm used in classical machine learning. Consider the cost function

$$C(s) = \frac{1}{N}\sum_{x=1}^{N}\langle\phi_x^{\text{out}}|\rho_x^{\text{out}}(s)|\phi_x^{\text{out}}\rangle.$$

To evaluate the derivative of the cost function, we will translate the formula for $\mathrm{d}C(s)/\mathrm{d}s$ (to order ϵ) from (3) to the channel formalism:

$$\begin{aligned}
\frac{\mathrm{d}C(s)}{\mathrm{d}s} &= \frac{i}{N}\sum_x \mathrm{tr}\Big(\mathbb{I}_{\text{in,hidden}}\otimes|\phi_x^{\text{out}}\rangle\langle\phi_x^{\text{out}}|\ \Big(\Big[K_{m_{\text{out}}}^{\text{out}}(s), U_{m_{\text{out}}}^{\text{out}}(s)\ldots U_1^1(s)\left(\rho_x^{\text{in}}\otimes|0\ldots0\rangle_{\text{hidden,out}}\langle0\ldots0|\right)U_1^{1\dagger}(s)\\
&\quad\ldots U_{m_{\text{out}}}^{\text{out}\ \dagger}(s)\Big] + \cdots + U_{m_{\text{out}}}^{\text{out}}(s)\ldots U_2^1(s)\left[K_1^1(s), U_1^1(s)\left(\rho_x^{\text{in}}\otimes|0\ldots0\rangle_{\text{hidden,out}}\langle0\ldots0|\right)U_1^{1\dagger}(s)\right]\\
&\quad U_2^{1\dagger}(s)\ldots U_{m_{\text{out}}}^{\text{out}\ \dagger}(s)\Big)\Big)\\
&= \frac{i}{N}\sum_{x=1}^{N}\sum_{l=1}^{L+1}\sum_{j=1}^{m_l}\mathrm{tr}\Big(U_1^{l+1\dagger}(s)\ldots U_{m_{\text{out}}}^{\text{out}\ \dagger}(s)\left(\mathbb{I}_L\otimes|\phi_x^{\text{out}}\rangle\langle\phi_x^{\text{out}}|\right)U_{m_{\text{out}}}^{\text{out}}(s)\ldots U_1^{l+1}(s)\\
&\quad U_{m_j}^l(s)\ldots U_{j+1}^l(s)\left[K_j^l(s), U_j^l(s)\ldots U_1^l(s)\left(\rho_x^{l-1}\otimes|0\ldots0\rangle_l\langle0\ldots0|\right)U_1^{l\dagger}(s)\ldots U_j^{l\dagger}(s)\right]U_{j+1}^{l\ \dagger}(s)\ldots U_{m_j}^{l\ \dagger}(s)\Big)\\
&= \frac{i}{N}\sum_{x=1}^{N}\sum_{l=1}^{L+1}\mathrm{tr}\Big(\mathcal{F}_s^{l+1}\left(\ldots\mathcal{F}_s^{\text{out}}\left(|\phi_x^{\text{out}}\rangle\langle\phi_x^{\text{out}}|\right)\ldots\right)\\
&\quad\sum_{j=1}^{m_j}U_{m_j}^l(s)\ldots U_{j+1}^l(s)\left[K_j^l(s), U_j^l(s)\ldots U_1^l(s)\left(\rho_x^{l-1}\otimes|0\ldots0\rangle_l\langle0\ldots0|\right)U_1^{l\dagger}(s)\ldots U_j^{l\dagger}(s)\right]U_{j+1}^{l\ \dagger}(s)\ldots U_{m_j}^{l\ \dagger}(s)\Big)\\
&= \frac{1}{N}\sum_{x=1}^{N}\sum_{l=1}^{L+1}\mathrm{tr}\left(\sigma_x^l(s)\mathcal{D}_s^l\left(\rho_x^{l-1}(s)\right)\right),
\end{aligned}$$

where $\sigma_x^l(s) = \mathcal{F}_s^{l+1}\left(\ldots\mathcal{F}_s^{\text{out}}\left(|\phi_x^{\text{out}}\rangle\langle\phi_x^{\text{out}}|\right)\ldots\right)$ and $\mathcal{D}_s^l = \partial\mathcal{E}_s^l/\partial s$ is the derivative of the corresponding channel, calculated by

$$\mathcal{D}_s^l\left(X^{l-1}\right) = \sum_{j=1}^{m_j} U_{m_j}^l(s)\ldots U_{j+1}^l(s)\left[K_j^l(s), U_j^l(s)\ldots U_1^l(s)\left(\rho_x^{l-1}\otimes|0\ldots0\rangle_l\langle0\ldots0|\right)U_1^{l\dagger}(s)\ldots U_j^{l\dagger}(s)\right]U_{j+1}^{l\;\dagger}(s)\ldots U_{m_j}^{l\;\dagger}(s)$$

and $\mathcal{F}_s^l$ being the adjoint channel of $\mathcal{E}_s^l$. The formula for $M_j^l(s)$ in the training algorithm (equation) simplifies to

$$M_j^l(s) = \left[U_j^l(s)\ldots U_1^l(s)\left(\rho_x^{l-1}(s)\otimes|0\ldots0\rangle_l\langle0\ldots0|\right)U_1^{l\dagger}(s)\ldots U_j^{l\dagger}(s), U_{j+1}^{l\;\dagger}(s)\ldots U_{m_l}^{l\;\dagger}(s)\left(\mathbb{I}_l\otimes\sigma_x^l(s)\right)U_{m_l}^l(s)\ldots U_{j+1}^l(s)\right].$$

It will we be useful for the implementation of the network to have an explicit expression of the adjoint channel $\mathcal{F}_s^l$. In order to obtain this we write the channel $\mathcal{E}_s^l$ in its Kraus representation, which is for any operator X^{l-1} on the $(l-1)$th layer

$$\mathcal{E}_s^l(X^{l-1}) = \sum_\alpha A_\alpha X^{l-1}A_\alpha^\dagger.$$

Here we have omitted the indices s and l for the Kraus operators A_α to make the notation clearer. Note that each of the Kraus operators A_α is a map from the $(l-1)$th layer consisting of m_{l-1} qubits to the lth layer consisting of m_l qubits. The adjoint channel $\mathcal{F}_s^l$ is then by definition given by

$$\mathcal{F}_s^l(X^l) = \sum_\alpha A_\alpha^\dagger X^l A_\alpha, \tag{5}$$

for any operator X^l on the lth layer.

We are now seeking for an explicit formula of the Kraus operators A_α. Let $\{|\alpha\rangle\}_\alpha$ be an orthonormal basis in the $(l-1)$th layer. Moreover, let $|m\rangle, |n\rangle$ be any vectors in the $(l-1)$th layer and $|i\rangle, |j\rangle$ any vectors in the lth layer. Then the action of $\mathcal{E}_s^l$ can be calculated using (4) and the shorthand notation $U^l(s) = U_{m_l}^l(s)\ldots U_1^l(s)$ for the whole unitary of the layer l, which gives

$$\begin{aligned}\langle i|\,\mathcal{E}_s^l\left(|m\rangle\langle n|\right)|j\rangle &= \left\langle i\middle|\,\text{tr}_{l-1}\left(U^l(s)\left(|m\rangle\langle n|\otimes|0\ldots0\rangle_l\langle0\ldots0|_l\right)U^{l\dagger}(s)\right)\middle|j\right\rangle\\ &= \sum_\alpha\left\langle\alpha,i\middle|\,U^l(s)\left(|m\rangle\langle n|\otimes|0\ldots0\rangle_l\langle0\ldots0|_l\right)U^{l\dagger}(s)\middle|\alpha,j\right\rangle\\ &= \sum_\alpha\left\langle\alpha,i\middle|\,U^l(s)\middle|m,0\ldots0\right\rangle\left\langle n,0\ldots0\middle|U^{l\dagger}(s)\middle|\alpha,j\right\rangle.\end{aligned}$$

Therefore, defining A_α via $\langle i|\,A_\alpha\,|m\rangle = \left\langle\alpha,i\middle|\,U^l(s)\middle|m,0\ldots0\right\rangle$ this gives a set Kraus operators for $\mathcal{E}_s^l$. Using this definition and (5) we obtain

$$\begin{aligned}\langle m|\,\mathcal{F}_s^l(|i\rangle\langle j|)\,|n\rangle = \sum_\alpha\langle m|\,A_\alpha^\dagger\,|i\rangle\langle j|\,A_\alpha\,|n\rangle &= \sum_\alpha\left\langle m,0\ldots0\middle|\,U^{l\dagger}(s)\middle|\alpha,i\right\rangle\left\langle\alpha,j\middle|U^l(s)\middle|n,0\ldots0\right\rangle\\ &= \left\langle m,0\ldots0\middle|\,U^{l\dagger}(s)\left(\mathbb{I}_{l-1}\otimes|i\rangle\langle j|\right)U^l(s)\middle|n,0\ldots0\right\rangle\\ &= \left\langle m\middle|\,\text{tr}_l\left(\mathbb{I}_{l-1}\otimes|0\ldots0\rangle_l\langle0\ldots0|_l U^{l\dagger}(s)\left(\mathbb{I}_{l-1}\otimes|i\rangle\langle j|\right)U^l(s)\right)\middle|n\right\rangle.\end{aligned}$$

From this we already know the action of $\mathcal{F}_s^l$ on a general operator X^l, which is

$$\mathcal{F}_s^l(X^l) = \text{tr}_l\left(\mathbb{I}_{l-1}\otimes|0\ldots0\rangle_l\langle0\ldots0|_l U^{l\dagger}(s)\left(\mathbb{I}_{l-1}\otimes X^l\right)U^l(s)\right).$$

SUPPLEMENTARY NOTE 3: NOISY NEURONS

Current NISQ devices are by definition noisy. Moreover, the fidelity and, consequently, cost function can be measured only with finite precision. Thus, it is important to examine if the QNN can still be useful if implemented on realistic devices.

We model the noise in the network by evolving the state with a random time-dependent Hamiltonian $H(\tau)$ before and after each operation. As a result the noisy unitary that corresponds to jth neuron in lth layer is

$$U_j^l(t) = R(t) U_j^l \tilde{R}(t), \tag{6}$$

$R(t)$ and $\tilde{R}(t)$ are two different realisations of a random unitary generated by the same probabilistic process. The update rule is also modified as

$$U_j^l(t) \to R(t) \left(e^{i\epsilon K_j^l} U_j^l \right) \tilde{R}(t). \tag{7}$$

The evolution with $H(\tau)$ can be constructed via a quantum Brownian circuit [4, 5]. Let us consider a family of Hamiltonians $\{H_j = H(j\Delta\tau)\}_{j=1}^n$, $\Delta\tau = T/n$, every H_j is Hermitian and its entries are Gaussian distributed with zero mean and a standard deviation of $\frac{2\pi\hbar\nu}{\sqrt{2^m n}}$. The noise strength is captured by a dimensionless parameter $t = \nu T$. We model both $R(t)$ as

$$R(t) = \prod_{j=1}^{n} \exp(i H_j \Delta\tau/\hbar) \,. \tag{8}$$

By Itô's calculus, there exists $H(t)$ such that

$$R(t) = \mathcal{T} \exp\left(i/\hbar \int_0^T H(\tau)\, \mathrm{d}\tau \right) + O\left(\frac{1}{\sqrt{n}} \right), \tag{9}$$

where $\mathcal{T}$ is the time ordering operator. We have used $n = 20$ for the calculations depicted in the main paper.

In Fig.2 in the main paper, the results of training a noisy QNN are depicted. We trained it for the task of generalisation and have also studied the robustness of the noisy network to corrupted data as described in the main paper. We studied this for variable noise strengths. We observe that results are close to the ideal QNN for small enough noise, moreover, learning is even more successful if a large fraction of input data is noisy. Fidelities required for successful learning are within reach of current quantum computers (see Supplementary Figure 3 and compare with [6, 7]).

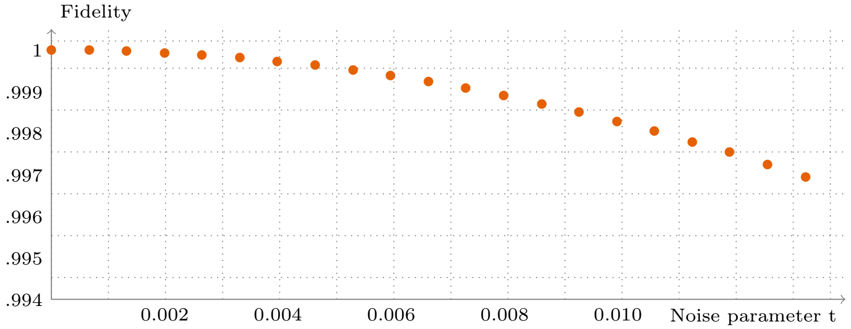

Supplementary Figure 3: The relation between fidelity and noise strength. The fidelity is computed for a single perceptron that maps a state to itself under the influence of noise. We have used a training set of 500 pairs for this calculation.

SUPPLEMENTARY NOTE 4: QUANTUM ALGORITHM FOR QUANTUM TRAINING OF THE NEURAL NETWORK

In this section we explain how our algorithm can be implemented on a quantum computer. To begin we want to clarify what operations a quantum computer is assumed to be able to do in our case:

1. Partial trace.

2. Initialize a qubit in $|0\rangle$ state.

3. Apply CNOT, T, H (and therefore perceptrons) easily. (The Solovay-Kitaev theorem says that any 2-qubit unitary can be built out of $O(\log^c(\frac{1}{\epsilon}))$ gates, where ϵ is the accuracy [2]).

4. Measuring in computational basis.

From now on we have two tasks. We need to compute the cost function as well as work out the derivative of the cost function on a quantum computer. We label/describe these two tasks as subroutine 1 and subroutine 2, respectively.

Subroutine 1. In this subroutine we use the "SWAP trick" to estimate the fidelity of a pure state $|\phi\rangle$ with a mixed state ρ. Our input is the state $|\phi\rangle$ in a register of m qubits and ρ in another register of m qubits. In total we have $2m$ qubits, however, we require an additional ancillary qubit for the following process. We estimate $F(|\phi\rangle, \rho) = \langle\phi|\rho|\phi\rangle$ as a probability exploiting the following quantum circuit.

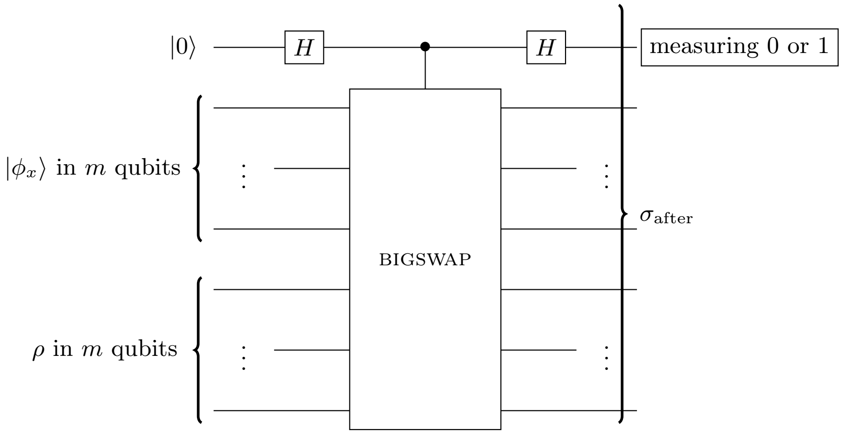

Supplementary Figure 4: Quantum circuit for computing the cost function.

To explain our subroutine we assume $m = 1$ for simplicity.

1a. **Initialization:** We initialize the $2m + 1$ qubits in the state

$$|0\rangle\langle 0| \otimes |\phi\rangle\langle\phi| \otimes \rho.$$

1b. **Hadamard:** In the next step we apply the Hadamard gate and end up with the state

$$\frac{1}{2}(|0\rangle + |1\rangle)(\langle 0| + \langle 1|) \otimes |\phi\rangle\langle\phi| \otimes \rho.$$

1c. **CSWAP:** We use $\text{CSWAP} := |0\rangle\langle 0| \otimes \mathbb{1} + |1\rangle\langle 1| \otimes \text{SWAP}$ and the result is

$$\begin{aligned}
&\text{CSWAP}^\dagger \left(\frac{1}{2}(|0\rangle + |1\rangle)(\langle 0| + \langle 1|) \otimes |\phi\rangle\langle\phi| \otimes \rho\right) \text{CSWAP} \\
&= \frac{1}{2}|0\rangle\langle 0| \otimes |\phi\rangle\langle\phi| \otimes \rho + \frac{1}{2}|1\rangle\langle 0| \left(\text{SWAP}(|\phi\rangle\langle\phi| \otimes \rho)\right) + \frac{1}{2}|0\rangle\langle 1| \left((|\phi\rangle\langle\phi| \otimes \rho)\,\text{SWAP}\right) \\
&+ \frac{1}{2}|1\rangle\langle 1| \left(\text{SWAP}(|\phi\rangle\langle\phi| \otimes \rho)\,\text{SWAP}\right).
\end{aligned}$$

1d. **Hadamard:** After applying the Hadamard gate a second time we have the following expression:

$$\begin{aligned}
\sigma_{\text{after}} =&\frac{1}{4}(|0\rangle + |1\rangle)(\langle 0| + \langle 1|) \otimes (|\phi\rangle\langle\phi| \otimes \rho) + \frac{1}{4}(|0\rangle - |1\rangle)(\langle 0| + \langle 1|) \otimes (\text{SWAP}(|\phi\rangle\langle\phi| \otimes \rho)) \\
&+ \frac{1}{4}(|0\rangle + |1\rangle)(\langle 0| - \langle 1|) \otimes ((|\phi\rangle\langle\phi| \otimes \rho)\,\text{SWAP}) + \frac{1}{4}(|0\rangle - |1\rangle)(\langle 0| - \langle 1|) \otimes (\text{SWAP}(|\phi\rangle\langle\phi| \otimes \rho)\,\text{SWAP}).
\end{aligned}$$

1e. **Measuring:** In this last step we measure the first control qubit and get 0 with probability p_0:

$$\begin{aligned}
p_0 =& \operatorname{tr}(|0\rangle\langle 0| \otimes \mathbb{1} \otimes \mathbb{1} \times \sigma_{\text{after}}) \\
=& \frac{1}{4}\operatorname{tr}(|0\rangle\langle 0| \big((|0\rangle + |1\rangle)(\langle 0| + \langle 1|)\big)\operatorname{tr}(|\phi\rangle|\phi\rangle \otimes \rho) \\
&+ \frac{1}{4}\operatorname{tr}(|0\rangle\langle 0| \big((|0\rangle - |1\rangle)(\langle 0| + \langle 1|)\big)\operatorname{tr}\left(\text{SWAP}(|\phi\rangle\langle\phi| \otimes \rho)\right) \\
&+ \frac{1}{4}\operatorname{tr}(|0\rangle\langle 0| \big((|0\rangle + |1\rangle)(\langle 0| - \langle 1|)\big)\operatorname{tr}\left((|\phi\rangle\langle\phi| \otimes \rho)\,\text{SWAP}\right) \\
&+ \frac{1}{4}\operatorname{tr}(|0\rangle\langle 0| \big((|0\rangle - |1\rangle)(\langle 0| - \langle 1|)\big)\operatorname{tr}\left(\text{SWAP}(|\phi\rangle\langle\phi| \otimes \rho)\,\text{SWAP}\right) \\
=& \frac{1}{4} + \frac{1}{4}\operatorname{tr}(\text{SWAP}\,|\phi\rangle\langle\phi| \otimes \rho) + \frac{1}{4}\operatorname{tr}(|\phi\rangle\langle\phi| \otimes \rho\,\text{SWAP}) + \frac{1}{4} \\
=& \frac{1}{2} + \frac{1}{2}\operatorname{tr}(\text{SWAP}\,|\phi\rangle\langle\phi| \otimes \rho).
\end{aligned}$$

Using the definition $\text{SWAP} = \sum_{j,k=1}^{2} |jk\rangle\langle kj|$ we obtain:

$$\begin{aligned}
\frac{1}{2} + \frac{1}{2}\operatorname{tr}(\text{SWAP}\,|\phi\rangle\langle\phi| \otimes \rho) =& \frac{1}{2} + \frac{1}{2}\sum_{j,k}\operatorname{tr}(|jk\rangle\langle kj|\,(|\phi\rangle\langle\phi| \otimes \rho) \\
=& \frac{1}{2} + \frac{1}{2}\sum_{j,k}\langle k|\phi\rangle\langle j|\rho|k\rangle\langle\phi|j\rangle \\
=& \frac{1}{2} + \frac{1}{2}\sum_{j,k}\langle\phi|j\rangle\langle j|\rho|k\rangle\langle k|\phi\rangle \\
=& \frac{1}{2} + \frac{1}{2}F\left(|\phi\rangle, \rho\right).
\end{aligned}$$

At this point we encounter quantum projective noise, i.e., we get 0 or 1 randomly and need to repeat this measurement N times to reduce the fluctuations arising from the binomial probability distribution. We get

$$\frac{\#0\text{s}}{N} = p_0 + \delta p_0$$
$$\frac{\#1\text{s}}{N} = p_1 + \delta p_1$$

with fluctuations $\delta p_i = \sqrt{\frac{p_i(1-p_i)}{N}} \approx \sqrt{\frac{p_i}{N}}$. Our resource usage so far amounts to:

- $2N$ Hadamards,
- N copies of $|\phi\rangle$,
- N copies of ρ, and
- N CSWAPs.

In addition to that we need m qubits for the operation

$$\text{CSWAP} = |0\rangle\langle 0| \otimes \mathbb{1} \otimes \mathbb{1} + |1\rangle\langle 1| \otimes \text{BIGSWAP},$$

where

$$\text{BIGSWAP} = \sum_{j_1,\dots j_m;k_1,\dots,k_m} |j_1,\dots,j_m;k_1,\dots,k_m,\rangle\langle k_1,\dots,k_m;j_1,\dots,j_m|.$$

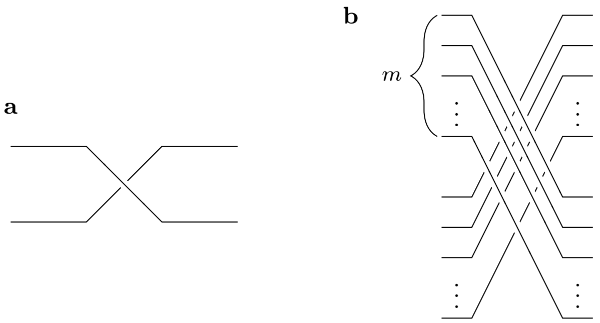

Supplementary Figure 5: Depiction of gates. (**a**) shows the SWAP gate, (**b**) shows the BIGSWAP gate.

For BIGSWAP m^2 swaps are needed if we arrange the qubits on a line, or m swaps otherwise. This concludes the description of our first subroutine.

To complete the description of our quantum algorithm we need to estimate the derivative of the cost function. This can be achieved by exploiting the following subroutine.

Subroutine 2. Subroutine 2 implements the channel $\mathcal{E}^l$. This part of the algorithm takes as input m_{l-1} qubits in the state ρ^{l-1}, where m_{l-1} is the number of qubits in layer $l-1$. The output is $\rho^l = \mathcal{E}^l(\rho^{l-1})$.

2a. **Initialization**
Tensor m_l qubits in state $|0\rangle$ with the input:

$$\rho^{l-1} \to \rho^{l-1} \otimes \underbrace{|0\rangle\langle 0| \otimes \ldots \otimes |0\rangle\langle 0|}_{m_l}.$$

Recources: In this step $m_{l-1} + m_l$ qubits are required.

2b. **Perceptrons**
Apply the perceptrons in layer l:

$$\rho^{l-1} \otimes |0\ldots 0\rangle\langle 0\ldots 0| \to \left(\prod_{k=1}^{n_l} U_k^l\right) \rho^{l-1} \otimes |0\ldots 0\rangle\langle 0\ldots 0| \left(\prod_{k=1}^{n_l} U_k^l\right)^\dagger = \tilde{\rho}^{l-1,l}.$$

Resources: We require $m_{l-1} + m_l$ qubits and n_l gates.

2c. **Partial trace**
Take the partial trace over layer m_{l-1}:

$$\tilde{\rho}^{l-1,l} \xrightarrow{\text{tr}} \rho^l.$$

Resources: In this step we go from $m_{l-1} + m_l$ qubits to m_l qubits without any gates.

To get ρ_{out} from ρ_{in} we need to repeat Steps $2a$ to $2c$ a total of L times. The total number of qubits required to carry out this subroutine is given by $\max\{m_1 + m_2, m_2 + m_3, \ldots, m_L + m_{\text{out}}\}$. We need to apply $n_1 + n_2 + \ldots + n_L$ perceptrons, where n_i is the number of perceptrons in layer i.

Algorithm for calculating the cost function. Putting it all together we can estimate the cost function via three steps:

1. Prepare 2 copies of the state $|\phi_x\rangle$ with probability $\frac{1}{N}$.

2. Do subroutine 2 on the last m qubits.

3. Do SWAP trick.

4. Repeat Steps 1,2, and 3 a total of M times for same value of x to estimate $\langle\phi_x|\rho|\phi_x\rangle$. (The choice of M affects the accuracy of the latter; the bigger M the more accurate we get.)

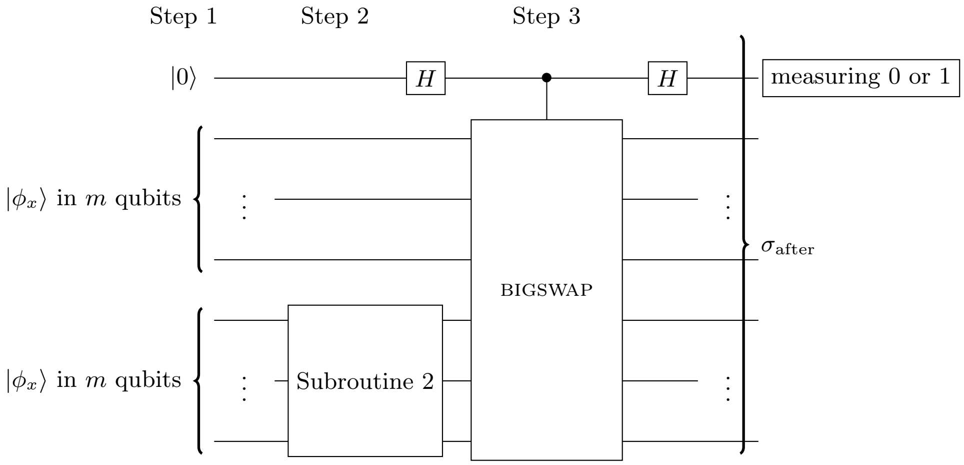

Supplementary Figure 6: Steps 1 to 3 of the algorithm for calculating the cost function.

Choose x randomly N times and employ this algorithm each time to compute the expectation value over x and thus the cost function $C = \frac{1}{N}\sum_x \langle\phi_x|\rho|\phi_x\rangle$. The total number of gates and perceptrons required is $N \times M(\sum_{i=1}^{L} n_i + 3)$. The number of qubits required is $\leq 2 \times W + m + 1$, where W is the width of the QNN, i.e., $W = \max\{m_1, \ldots, m_{\text{out}}\}$.

Algorithm for calculating the derivative. To work out the derivative $\frac{dC}{ds}$ of the cost function we compute $\frac{\delta C}{\delta y^\mu}$, where y^μ is the vector of all the parameters. For a single three-qubit perceptron $U = e^{ik}$ with $k = \sum k_{\alpha,\beta,\gamma}\sigma^\alpha \otimes \sigma^\beta \otimes \sigma^\gamma$ we write

$$y^\mu = \left.\begin{pmatrix} k_{000} \\ k_{001} \\ k_{002} \\ k_{003} \\ k_{010} \\ \vdots \end{pmatrix}\right\} = 64 \text{ parameters.}$$

For a four-qubit QNN with two three-qubit perceptrons, see Fig. ??, we have

$$y^\mu = \left.\begin{pmatrix} k^1_{000} \\ \vdots \\ k^1_{333} \\ k^2_{000} \\ \vdots \\ k^2_{333} \end{pmatrix}\right\} = 2 \times 64 \text{ parameters.} \tag{10}$$

Now we need to work out $\frac{\delta C}{\delta y^\mu} \approx \frac{C(y+\epsilon^\mu)-C(y)}{\epsilon}$, where

$$\epsilon^\mu = \begin{pmatrix} 0 \\ \vdots \\ 0 \\ \epsilon \\ 0 \\ \vdots \\ 0 \end{pmatrix},$$

i.e. ϵ is the αth entry and $\mu = 1, \ldots, (\#\text{perc}) \times 64$, where #perc denotes the numbers of perceptrons.

Suppose we know $C(y)$ and work out $\frac{\delta C}{\delta y^\mu}$, $Q = (\#\text{perc}) \times 64$ times. This gives us

$$y = \begin{pmatrix} \frac{\delta C}{\delta y^1} \\ \frac{\delta C}{\delta y^2} \\ \vdots \\ \frac{\delta C}{\delta y^Q} \end{pmatrix}.$$

All that is left to do is the gradient ascent step, with

$$y_{\text{new}} = y_{\text{old}} + \frac{1}{2\lambda} \begin{pmatrix} \frac{\delta C}{\delta y^1} \\ \frac{\delta C}{\delta y^2} \\ \vdots \\ \frac{\delta C}{\delta y^Q} \end{pmatrix}.$$

Using this and Taylor's theorem, we obtain an expression for the cost function at y_{new}:

$$\begin{aligned} C(y_{\text{new}}) &= C(y_{\text{old}} + \frac{1}{2\lambda}\frac{\delta C}{\delta y}) \\ &= C(y_{\text{old}}) + \sum_{\mu=1}^{Q} \frac{1}{2\lambda} \left(\frac{\delta C}{\delta y^\mu}(y_{\text{old}}) \right)^2 + \mathcal{O}(1/\lambda^2) \end{aligned}$$

Choosing now λ big enough, this shows that updating the parameters in the above way always makes the cost funtion larger, i.e.

$$C(y_{\text{new}}) - C(y_{\text{old}}) \geq 0.$$

SUPPLEMENTARY NOTE 5: IMPLEMENTATION DETAILS

Here, we compare our QNN training method with that of executing full state tomography of the intermediate states of the network, and then classically calculating the K matrices.

First, we need to estimate the numper of copies required by the quantum algorithm. The number of copies N_{copies} of each pair in the training set used in each round is large, as can be seen from the following formula:

$$N_{\text{copies}} = n_{\text{proj}} \times n_{\text{params}}. \tag{11}$$

Here n_{proj} is the factor coming from repetition of measurements to reduce projection noise (i.e., estimate expectation values via measurement), and n_{params} is the total number of parameters in the network given by

$$\begin{aligned} n_{\text{params}} &= \sum_{l=1}^{L+1} \sum_{j=1}^{m_l} \#\ \text{parameters}(U_j^l) \\ &= \sum_{l=1}^{L+1} \sum_{j=1}^{m_l} (4^{(m_{l-1}+1)} - 1) \\ &= \sum_{l=1}^{L+1} m_l \times (4^{(m_{l-1}+1)} - 1). \end{aligned} \tag{12}$$

U_j^l are the perceptron unitaries, and the index $L+1$ refers to the outgoing layer (i.e., U_j^{out}). To get the second line, we used that the number of parameters in the perceptrons in this work is given by $4^{(m_{l-1}+1)} - 1$. Recall that m_l is the number of perceptron unitaries acting on perceptrons in layer $l-1$ and layer l, and the -1 term occurs because the overall phase of the unitaries is unimportant.

As an example, let us ask how many copies of each training pair we would need to perform the quantum training of the network in Fig.2 in the paper. In that case, $n_{\text{params}} = 699$, and there were 300 rounds of training with 100 pairs. (Note that we actually need much fewer training pairs to train the network. The point in that part of the discussion

was to test how robust the training is when some pairs are noisy. For our purposes 8 will suffice.) Then we have that the total number of copies needed for training is

$$\begin{aligned} N_{\text{total copies}} &= n_{\text{proj}} \times n_{\text{params}} \times n_{\text{pairs}} \times n_{\text{rounds}} \\ &= 500 \times 699 \times 8 \times 300 \\ &= 838,800,000. \end{aligned} \tag{13}$$

Here we chose $n_{\text{proj}} = 500$ to reduce the projection noise below ~ 0.03. This used the bound on fluctuations discussed in appendix G1: the measurement used to estimate the fidelity estimates probabilities p with fluctuations given by $\sqrt{p(1-p)/n_{\text{proj}}} \leq 1/\sqrt{2n_{\text{proj}}}$.

This estimate of $N_{\text{total copies}}$ is overkill to a large extent. If we wanted to actually trial such a process on a quantum computer, we wouldn't train for 300 rounds, and, especially for noisy systems without error correction, there would be less need to choose n_{proj} so large. This is because the system would be noisy anyway. The interesting point in that case would be to test how much the circuit can learn on a noisy system more as a proof of principle.

Another straightforward way to reduce the required resources is to exploit sparsely connected QNNs.

We now look at a few ideas using state tomography to improve the training of our QNN.

(i) One option along these lines, is to use state tomography to find σ_x^l and ρ_x^{l-1} for a given l. From that we can find M_j^l, which will allow us to do the update. To do that we would need to simulate the evolution of $\rho_x^{l-1} \otimes |00...0\rangle_l\langle 00...0|$ and $I_{l-1} \otimes \sigma_x^l$ under the unitaries for those layers. However, the neural network should hopefully be useful in a regime where it cannot be easily classically simulated, so this would probably not be a feasible strategy.

(ii) We can consider an alternative use of the state tomography idea: to do the unitary updates in the second equation in point 3a and *then* do tomography to find the states, e.g., $\prod_{\alpha=j}^{1} U_\alpha^l (\rho_x^{l-1} \otimes |00...0\rangle_l\langle 00...0|) \prod_{\alpha=1}^{j} U_\alpha^{l\dagger}$. But then we would still have to classically calculate the commutator, which would be a difficult task for such large matrices.

Still, we can count how many copies $N_{\text{copies}}^{\text{tom}}$ of each pair in the training set (per round of training) we would need in this case.

$$N_{\text{copies}}^{\text{tom}} = n_{\text{proj}} \times \sum_{l=1}^{L+1} m_l \times 2[(d_l \times d_{l-1})^2 - 1]. \tag{14}$$

Here n_{proj} is again the factor coming from repetition of measurements to reduce projection noise (i.e., estimate expectation values via measurement), which we also need when doing measurements for state tomography. Then we sum over the layers with the summand $m_l \times 2[(d_l \times d_{l-1})^2 - 1]$. Here $d_l \times d_{l-1}$ arises because the states we look at live in $d_l \times d_{l-1}$-dimensional Hilbert spaces. The power of two follows from the usual reasoning that state tomography requires $O(d^2-1)$ measurements to characterize a state on a d-dimensional Hilbert space. The factor of 2 comes from the fact that we are doing this for two states, and finally the factor of m_l comes from the fact that there are m_l matrices M_j^l that we need to calculate for a given layer. Note that, if there were some additional information, we might be able to reduce the cost of the state tomography via, e.g., compressed sensing.

So the question now is how this value ($N_{\text{copies}}^{\text{tom}}$) compares to our value for N_{copies} from our usual approach. To make things simple, let's assume a network of qubits with constant width $m_l = m$ for all l. To compare $N_{\text{copies}}^{\text{tom}}$ and N_{copies} we can ignore factors of n_{proj} and just look at the summands (as the sums are over the same ranges: all layers). Then, because

$$m_l \times 2[(d_l \times d_{l-1})^2 - 1] = 2m(4^m - 1)^2, \tag{15}$$

is bigger than

$$(4^{m+1} - 1) \times m \tag{16}$$

for $m \geq 2$, we see that $N_{\text{copies}}^{\text{tom}} \geq N_{\text{copies}}$, so the state tomography trick doesn't help us in this case. A caveat is that with some useful ansatz about the states, some more specialized forms of state tomography may give us an advantage and $N_{\text{copies}}^{\text{tom}} \leq N_{\text{copies}}$, which could aid with training specialized networks. This is highly interesting and would be a good point for future work.

(iii) A final possibility is to use a modified version of a trick from [8]. In that case, we can encode the commutator of two states into the state of the system. Let's see how this works. Given two states ρ_A and σ_B of the

same dimension, we evolve the state of A and B, i.e., $\rho_A \otimes \sigma_B$, via the unitary $\exp(-iS\pi/4)$, where S is the swap operator. Then we trace out system A:

$$\mathrm{tr}_A[e^{-iS\pi/4}\rho_A \otimes \sigma_B\, e^{iS\pi/4}] = \frac{\rho_B + \sigma_B}{2} - \frac{i}{2}[\rho_B, \sigma_B], \tag{17}$$

where we used that $e^{-iSt} = \cos(t) - i\sin(t)S$, which follows because $S^2 = I$.

So we see that the commutator is encoded into the state of the B system. The update matrices K_j^l may be obtained from this expression by taking the partial trace, i.e., by measuring some local observables. To eliminate $(\rho_B + \sigma_B)/2$ we need to compute ρ_B and σ_B though. It might well be possible to make this approach coherent and eliminate completely the requirement that we have access to many copies of the training data; we will investigate this in a future paper. of the neural network, n_{params} can be reduced significantly.

---

[1] Wolf, M. M. Mathematical foundations of supervised learning. `https://www-m5.ma.tum.de/foswiki/pub/M5/Allgemeines/MA4801_2018S/ML_notes_main.pdf` (2018). [Online; accessed 25-February-2019].

[2] Nielsen, M. A. & Chuang, I. L. *Quantum Computation and Quantum Information* (Cambridge University Press, 2010).

[3] Werner, R. F. *Quantum Information Theory – an Invitation*, 14–57 (Springer Berlin Heidelberg, Berlin, Heidelberg, 2001).

[4] Zhou, T. & Chen, X. Operator dynamics in a brownian quantum circuit. *Phys. Rev. E* **99**, 052212 (2019).

[5] Lashkari, N., Stanford, D., Hastings, M., Osborne, T. & Hayden, P. Towards the fast scrambling conjecture. *J. High Energy Phys.* **2013**, 22 (2013).

[6] Barends, R. *et al.* Diabatic gates for frequency-tunable superconducting qubits. *arXiv:1907.02510* **123**, 210501 (2019).

[7] Levine, H. *et al.* High-fidelity control and entanglement of rydberg-atom qubits. *Phys. Rev. Lett.* **121** (2018). URL `http://dx.doi.org/10.1103/PhysRevLett.121.123603`.

[8] Lloyd, S., Mohseni, M. & Rebentrost, P. Quantum principal component analysis. *Nature Physics* **10**, 631–633 (2014).
